# A Protocol for Cortical Type Analysis of the Human Neocortex Applied on Histological Samples, the Atlas of Von Economo and Koskinas, and Magnetic Resonance Imaging

**DOI:** 10.3389/fnana.2020.576015

**Published:** 2020-12-07

**Authors:** Miguel Ángel García-Cabezas, Julia Liao Hacker, Basilis Zikopoulos

**Affiliations:** ^1^Department of Anatomy, Histology and Neuroscience, School of Medicine, Universidad Autónoma de Madrid, Madrid, Spain; ^2^Neural Systems Laboratory, Department of Health Sciences, Boston University, Boston, MA, United States; ^3^Human Systems Neuroscience Laboratory, Department of Health Sciences, Boston University, Boston, MA, United States; ^4^Department of Anatomy and Neurobiology, Boston University School of Medicine, Boston, MA, United States; ^5^Graduate Program in Neuroscience, Boston University, Boston, MA, United States

**Keywords:** Brodmann area, cortical area, cortical layer, structural model, cytoarchitecture, Nissl

## Abstract

The human cerebral cortex is parcellated in hundreds of areas using neuroanatomy and imaging methods. Alternatively, cortical areas can be classified into few cortical types according to their degree of laminar differentiation. Cortical type analysis is based on the gradual and systematic variation of laminar features observed across the entire cerebral cortex in Nissl stained sections and has profound implications for understanding fundamental aspects of evolution, development, connections, function, and pathology of the cerebral cortex. In this protocol paper, we explain the general principles of cortical type analysis and provide tables with the fundamental features of laminar structure that are studied for this analysis. We apply cortical type analysis to the micrographs of the Atlas of the human cerebral cortex of von Economo and Koskinas and provide tables and maps with the areas of this Atlas and their corresponding cortical type. Finally, we correlate the cortical type maps with the T1w/T2w ratio from widely used reference magnetic resonance imaging scans. The analysis, tables and maps of the human cerebral cortex shown in this protocol paper can be used to predict patterns of connections between areas according to the principles of the Structural Model and determine their level in cortical hierarchies. Cortical types can also predict the spreading of abnormal proteins in neurodegenerative diseases to the level of cortical layers. In summary, cortical type analysis provides a theoretical and practical framework for directed studies of connectivity, synaptic plasticity, and selective vulnerability to neurologic and psychiatric diseases in the human neocortex.

## Introduction

The microscopic structure of the cerebral cortex can be studied in Nissl-stained sections following two different epistemological and theoretical approaches. The most common approach identifies characteristic cytoarchitectonic features (or a combination of features) that are found in a given part of the cortex and absent in others. Such different and particular cytoarchitectonic features allow the distinction of cortical areas. For instance, a band of Betz cells in layer V is the hallmark of the primary motor cortex (Bevan Lewis and Clarke, 1878), area 4 of Brodmann (Brodmann, 1909/1999). Cortical areas defined with this approach were used for the construction of classical maps of the human cerebral cortex (Hammarberg, 1895; Campbell, 1905; Brodmann, 1909/1999; Vogt and Vogt, 1919; von Economo and Koskinas, 1925/2008; Bailey and Von Bonin, 1951; Sarkissov et al., 1955) and of the cortex of other primates (Von Bonin and Bailey, 1947) and rodents (Krieg, 1946; Caviness, 1975; Zilles, 1985). Modern studies of cortical areas combine classical cytoarchitecture as examined in Nissl-stained sections with quantitative image analysis, myeloarchitecture, immunohistochemistry, receptor binding, and brain imaging (Amunts and Zilles, 2001, 2015; Zilles and Palomero-Gallagher, 2017; Palomero-Gallagher and Zilles, 2019) and provide detailed atlases of the entire human cerebral cortex that can be used by the neuroscience community (Amunts et al., 2013; Ding et al., 2016).

In an alternative approach, the many cortical areas, more than 170 areas in some studies of the human cortex (Vogt and Vogt, 1919; Glasser et al., 2016), can be classified in few cortical types according to their degree of laminar elaboration. Cortical types were first described by von Economo and Koskinas in the human cortex based on “the constant variations that one observes in each of the layers in different regions” (von Economo and Koskinas, 1925/2008; von Economo, 1927/2009). In other words, instead of particular cytoarchitectonic features, the basis of cortical type analysis lays in the gradual and systematic variation of laminar features (like number of layers, prominence of layers, and sharpness of laminar boundaries) observed across the cortical quilt. Accordingly, different areas that are distinguished by their characteristic cytoarchitectonic features can show comparable degree of laminar elaboration and, thus, comparable cortical type. An important aspect of cortical type analysis is the topological distribution of these types across the cortical quilt. The laminar structure of the cortex varies across gradients in which one cortical type transitions gradually into another type without abrupt jumps from areas of poor laminar elaboration into areas of sharp laminar elaboration. Therefore, adjacent areas are either of the same type or of types with small increments or decrements of laminar elaboration. On the other hand, distant areas in different lobes and systems can have either comparable laminar structure or different cortical types, but cortical type transitions with adjacent areas will always respect the topological arrangement of gradients (Garcia-Cabezas et al., 2019).

The study of cortical types and systematic variation of laminar architecture across the cerebral cortex has profound implications for understanding fundamental aspects of cortical evolution, development, connections, function, and pathology (Garcia-Cabezas and Zikopoulos, 2019). For instance, research based on the cortical type approach showed that gradual and systematic variation of laminar structure across cortical areas and across mammalian species is rooted in evolution leading to the proposal of the Dual Origin of the Neocortex Hypothesis (Abbie, 1940, 1942; Sanides, 1970; Reep, 1984; Pandya et al., 1988, 2015; Garcia-Cabezas et al., 2019). This hypothesis is supported by recent developmental studies that provide organizers and molecular mechanisms for the tangential expansion of the cerebral cortex and the emergence of cortical types in development and, likely, in evolution (reviewed in Subramanian et al., 2009; Garcia-Cabezas et al., 2019; Puelles et al., 2019).

Cortical types are also related to cortical connections in a relational model called the Structural Model for Connections. According to this model, the laminar pattern of connections between two given neocortical areas can be predicted if the cortical types of these areas are known (Barbas, 1986, 2015; Barbas and Rempel-Clower, 1997; Garcia-Cabezas et al., 2019). Areas of the same cortical type in different lobes are also more likely connected by long range connections than areas of different type (Zikopoulos et al., 2018) and have neighboring territories of projection in the striatum (Yeterian and Pandya, 1991). The spread of cortico-cortical projections from one area across few or more areas also seems to be related to cortical type (Morecraft et al., 2004, 2012, 2015).

Regarding function, cortical types are related to the degree of synaptic plasticity, as shown by opposing trends in the expression of markers that favor plasticity or stability along gradients of laminar elaboration (Garcia-Cabezas et al., 2017). Cellular features, like dendrite length and dendrite complexity, also vary systematically in parallel to laminar differentiation (Elston et al., 2005, reviewed in García-Cabezas et al., 2018). Finally, cortical areas with the simplest laminar structure seem to be more vulnerable to neurodegenerative diseases, epilepsy, and neurodevelopmental disorders compared to areas with better laminar definition (Arnold et al., 1991; Braak and Braak, 1991; Duyckaerts et al., 1998; Zikopoulos and Barbas, 2010; Vismer et al., 2015; Burt et al., 2018; Zikopoulos et al., 2018).

Some studies of the human cerebral cortex have categorized cortical areas using the cortical type approach (Galaburda and Sanides, 1980; García-Cabezas et al., 2018; Zikopoulos et al., 2018), but systematic analysis of cortical types across the entire human cortex is lacking. In this protocol paper we explain the general principles of cortical type analysis using Nissl-stained sections of the human prefrontal cortex. We also study the laminar architecture of visual areas in the occipital lobe to explore koniocortical and parakoniocortical areas, two cortical types absent in the prefrontal cortex (Sanides, 1970). We provide tables with the fundamental features of laminar structure that are studied for cortical type analysis and show representative examples of each type in figures. Then, we apply this analysis to 100 neocortical areas, subareas and transitional zones between areas across the cortical surface depicted in high-quality micrographs in the Atlas of the human cerebral cortex of von Economo and Koskinas (1925/2008). We also provide tables with the names and abbreviations of areas across the human neocortex from the Atlas of von Economo and Koskinas (1925/2008) with their corresponding cortical type and show them on maps. Finally, we correlate the types represented in maps with three quantitative structural features of the cerebral cortex. First, we correlate cortical type with the T1w/T2w signal map (Glasser and Van Essen, 2011), which reflects the content of intracortical myelin and is widely used in imaging studies of cortical hierarchies and connectivity (Glasser et al., 2016; Zhang et al., 2020). Second, we measured the size of pyramidal neuron bodies in layers III and V because the presence of the largest pyramids across these layers is a major feature of laminar elaboration (Sanides, 1970; Goulas et al., 2018). Third, we compare the thickness in 43 areas of the human cortex measured by von Economo (1927/2009) with the results of cortical type analysis in the micrographs of the same 43 areas in the Atlas of von Economo and Koskinas (1925/2008) because thickness is an anatomical feature of cortical areas that is frequently measured by imaging techniques (e.g., Fischl and Dale, 2000; Scholtens et al., 2015; Wagstyl et al., 2020) and has been related to cortical hierarchies (Mahjoory et al., 2020).

The cortical type analysis described in this protocol paper will help predict patterns of connections between areas in the human neocortex and determine the level of these areas in cortical hierarchies. It can also predict the spreading of abnormal proteins in neurological disorders like Alzheimer’s disease to the level of cortical layers. Cortical type analysis also provides a framework for directed studies searching for factors of synaptic plasticity and selective vulnerability to neurologic and psychiatric diseases in the human neocortex.

## Materials and Equipment

### Human Post-mortem Brain Tissue: Fixation and Preparation

We analyzed the laminar structure of prefrontal and occipital cortical areas in Nissl-stained sections of neurotypical human *post-mortem* brain tissue (*n* = 4, cases MD12112758, VA12103176, ND14162, and ND11109) obtained from the National Disease Research Interchange (NDRI), and Anatomy Gifts Registry. The study was approved by the Institutional Review Board of Boston University. Data of human subjects are summarized in Table 1.

**TABLE 1:** Data of human subjects analyzed.

**Human subjects**	**Sex**	**Age (years)**	**Internal cat #**
MD12112758	F	58	HAW
VA12103176	F	67	HAY
ND14162	M	55	HCP
ND11109	M	38	HCD

**FIGURE 1:**
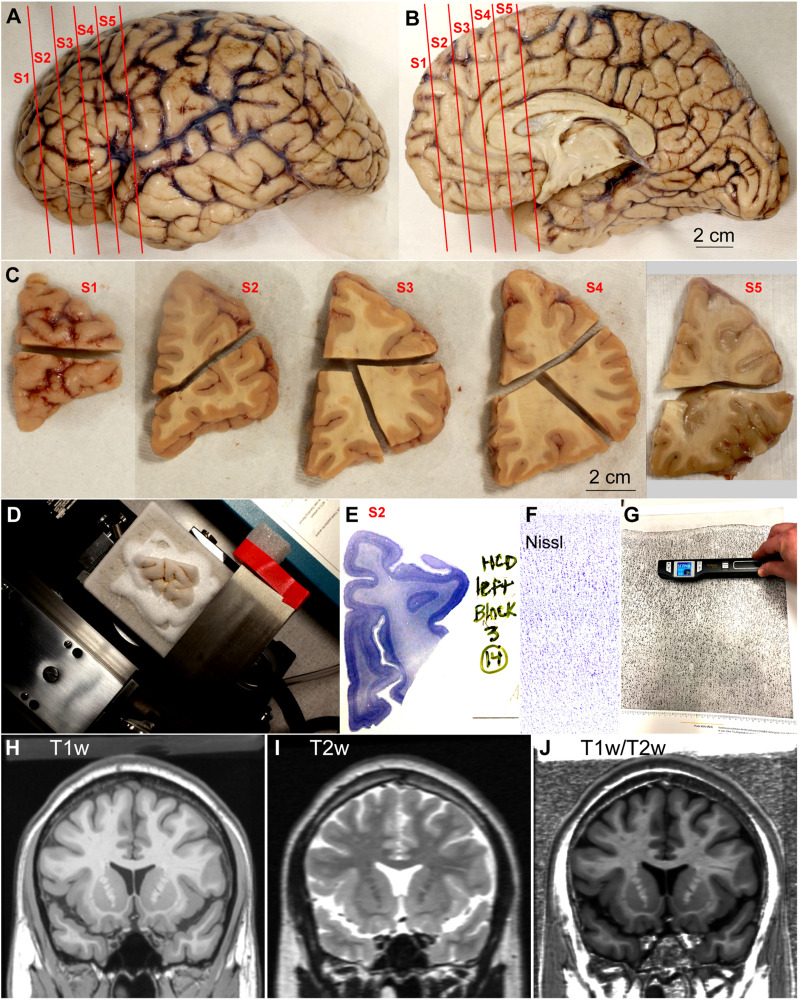
Outline of methods. **(A,B)** Lateral **(A)** and medial **(B)** views of the left hemisphere from the brain (case HCD): red lines show the planes of separation of coronal slabs. **(C)**, Slabs of the left hemisphere shown in **(A,B)** cut in smaller blocks; anterior is left and posterior is right. **(D)**, Dorsal block of S1 **(C)** frozen on the sliding microtome for cutting into series of sections. **(E)**, Section of dorsal block of S2 **(C)** stained for Nissl. **(F)**, Micrograph of a cortical area through the straight part of the gyrus from a Nissl stained section (original magnification: 10×). **(G)**, Scanning of micrograph from the Atlas of von Economo and Koskinas (1925/2008). **(H–J)**, Coronal MRI scans of the brain of T1-weighted signal, T2-weighted signal, and the ratio between T1 and T2-weighted signals. S1, S2, S3, S4, S5, indicate coronal slabs from anterior to posterior; T1w, T1-weighted scan; T2w, T2-weighted scan; T1w/T2w, T1/T2 ratio. Calibration bar in **(B)** applies to **(A,B)**.

Donated *post-mortem* human brains were immerse-fixed in 10% formalin. Brain peduncles were cut upon arrival in the Human Systems Neuroscience Laboratory at Boston University to separate the brainstem and cerebellum from the cerebral hemispheres; then, we cut the corpus callosum to separate the cerebral hemispheres and photographed their surface (basal, medial, lateral, and dorsal; Figures 1A,B) with a digital camera (Canon EOS 5N). We then sliced each cerebral hemisphere in coronal slabs of 1 cm thickness, photographed the anterior and posterior surfaces of each slab (Figure 1C) and post-fixed them in 10% formalin for 2–4 days. After post-fixation, we matched the slabs based on atlases of the human brain (von Economo and Koskinas, 1925/2008; Mai et al., 2015). Smaller blocks of the slabs containing prefrontal areas and occipital areas around the calcarine fissure were separated, photographed, cryoprotected in a series of sucrose solutions (10–30% in 0.01 M PBS), and frozen in −75^°^C isopentane (Thermo Fisher Scientific, Pittsburg, PA, United States) for rapid and uniform freezing (Rosene et al., 1986). Blocks were cut on a freezing microtome in the coronal plane at 50 μm (Figure 1D) and 10 consecutive series of sections were collected. Some blocks were not cryoprotected and, after embedding in agar (6%), were cut at 50 or 75 μm using a vibratome (PrecisionaryVF-700, Precisionary Instruments Inc., Greenville, NC, United States). Sections were stored in antifreeze solution (30% ethylene glycol, 30% glycerol, 40% PB 0.05 M at pH 7.4 with 0.05% azide) at −20^°^C for future assays.

### Nissl Staining

A series of sections of each block was mounted in gelatin coated slides (Gelatin Type A, G8-500, Fischer Chemicals, Fair Lawn, NJ, United States) and stained for Nissl (Figure 1E) as described in detail in a previous Protocol Paper (García-Cabezas et al., 2016). Briefly, sections on slides were dried, defatted in a 1:1 solution of chloroform and 100% ethanol for 1 h, rehydrated through a series of graded alcohols and distilled water, stained with 0.05% thionin (pH 4.5) for 3–5 min, differentiated through graded alcohols, defatted in xylene, and coverslipped with mounting media (Entellan, Electron Microscopy Sciences, Hatfield, PA, United States).

### SMI-32 Immunostaining

Selected sections of prefrontal blocks were processed for SMI-32, an antibody that labels a non-phosphorylated intermediate neurofilament protein. SMI-32 labels in the human cerebral cortex the neuron body and proximal dendrites of a subset of pyramidal projection neurons in layers III and V and, to a lesser extent, in layers II and VI (Campbell and Morrison, 1989). The density of neurons positive for SMI-32 varies across cortical areas and thus can be used as a sensitive architectonic marker (Hof et al., 1995; Barbas and García-Cabezas, 2015, 2016; García-Cabezas and Barbas, 2017).

Briefly, free-floating sections were rinsed in PBS (0.01 M at pH 7.4), incubated in glycine (0.05 M) and preblocked [20% normal goat serum (NGS), 20% bovine serum albumin (BSA), 2% BSA-C, and 0.2% Triton-X in PBS] for 1 h. Sections were then incubated during 48 h at 4°C in primary antibody against SMI-32 (mouse monoclonal, Sternberger Monoclonals, Lutherville, MD, United States; diluted 1:5,000 in PBS with 20% NGS, 20% BSA, 2% BSA-C, and 0.1% Triton-X), rinsed in PBS, and incubated for 3 h in secondary biotinylated goat antimouse IgG (Vector Laboratories, Burlingame, CA, United States; diluted 1:200 in PBS with 20% NGS, 20% BSA, 2% BSA-C, and 0.1% Triton-X), followed by 1 h in an avidin–biotin horseradish peroxidase complex (ABHRP; Vectastain PK-6100 ABC Elite kit, Vector Laboratories; diluted 1:100 in PBS). Sections were rinsed and processed for the peroxidase-catalyzed polymerization of diaminobenzidine (DAB; DAB kit, Vector Laboratories or Zymed Laboratories Inc., South San Francisco, CA, United States; 0.05% DAB, and 0.004% H_2_O_2_ in PBS) for 2–3 min under microscope control. After DAB development, sections were washed with PB (0.1 M, pH 7.35), mounted on gelatin-coated slides (Gelatin Type A, G8-500, Thermo Fisher Scientific), and coverslipped with mounting media (Entellan, Electron Microscopy Sciences).

### Optical Microscopy and Photography

Sections of the human prefrontal cortex stained for Nissl and SMI-32 and sections of visual areas in the occipital lobe stained for Nissl were examined with an optical microscope (Olympus, BX51) at low (2x: PlanApo 2x/0.08 Japan; 4x: UPlanFl 4x/0.13 Japan) and medium (10x: UPlanFL 10x/0.30 Japan) magnification. Micrographs (Tiff files) of cortical columns encompassing all layers were taken at 10x with a CCD camera (Olympus DP70) along straight (dome and gyral wall) and convex and concave turning gyral (dome to sulcal wall) and sulcal (in the fundus) parts sampling the entire cortical ribbon of the prefrontal region in Nissl stained coronal sections (Figure 1F). We also took micrographs of the primary visual area and adjacent areas (straight and turning). Micrographs were imported to PowerPoint software (Microsoft, Redmond, WA, United States) for blind analysis on a computer. Selected images were imported into Adobe Illustrator CC software (Adobe Systems Inc., San José, CA, United States) to assemble figures. Minor adjustment of overall contrast and brightness were made but the micrographs were not retouched.

### Scanning Micrographs of the Atlas of the Human Cerebral Cortex of von Economo and Koskinas (1925/2008)

For a complete analysis of the human cerebral cortex we used the Atlas of von Economo and Koskinas (1925/2008). The micrographs analyzed and shown in this atlas were taken from sections cut perpendicular to the long axis of straight parts of each area, either at the dome of the gyrus or at the gyral wall in the sulcus. We chose this Atlas because it covers the entire cerebral cortex and provides high quality micrographs and descriptions of the defined areas. Also, there is a digital version of this Atlas available for neuroimaging studies (Scholtens et al., 2018). We scanned the micrographs of neocortical areas with a hand scanner (Magic Wand^TM^ WI-FI^®^ II portable scanner, VuPoint Solutions, City of Industry, CA, United States; Figure 1G). We scanned the central portions of the micrographs to leave out the margins in which the authors labeled and identified cortical layers and sublayers. Tiff files of each micrograph scanned were obtained and imported to PowerPoint software for blind analysis on a computer.

### T1w/T2w MRI Signal Map of the Human Cerebral Cortex

To illustrate the practical use of cortical type we applied the results of cortical type analysis to MRI scans, commonly used in imaging studies. We used the T1-weighted (T1w; Figure 1H) and T2-weighted (T2w; Figure 1I) datasets of a public MRI scan [Colin 27 average brain dataset; stereotaxic registration model, high-resolution version 2008; Copyright (C) 1993–2009 Louis Collins, McConnell Brain Imaging Centre, Montreal Neurological Institute, McGill University] to obtain a map of the T1w/T2w ratio (Figure 1J) of the cerebral cortex. We estimated the T1w/T2w ratio using coronal, sagittal, and horizontal planes of the scans after reslicing with ImageJ (NIH, Bethesda, MD, United States). The T1w/T2w ratio has been used successfully to differentiate and map cortical areas (e.g., Glasser and Van Essen, 2011).

### Size of Pyramidal Neuron Bodies in Layers III and V in Relation to Cortical Type

We measured the size of pyramidal neuron bodies in layers III and V in micrographs of Nissl stained sections (cases HAW and HCD). Selected micrographs from representative areas (5 micrographs per area) of different cortical types (area LA_2_ in a region equivalent to Brodmann’s area 24a, area LD in a region equivalent to area prostriata, area FD*_*m*_* in a region equivalent to Brodmann’s area 46, and area OB, which is equivalent to Brodmann’s area 18) were open in ImageJ and calibrated. We then measured the area of the largest pyramids in layers III and V (∼200 neuron bodies per area) because the size of pyramidal neuron bodies in layers III and V is a major feature of laminar elaboration (Sanides, 1970; Goulas et al., 2018). Then, we divided the average size of the largest pyramidal neuron bodies in layer III by the average size of the largest pyramidal neuron bodies in layer V in each analyzed area to obtain a ratio (ratio = 1 when the size of pyramids is comparable in layers III and V; ratio < 1 when the largest pyramids are in layer V; and ratio > 1 when the largest pyramids are in layer III).

### Relation of Cortical Thickness and Cortical Type

We also compared cortical thickness measured for 43 areas of the human cortex by von Economo (1927/2009) with the results of cortical type analysis for the same areas in the micrographs of the Atlas of von Economo and Koskinas (1925/2008). Cortical thickness is an anatomical feature of cortical areas that is easy to measure by imaging techniques (e.g., Fischl and Dale, 2000; Scholtens et al., 2015; Wagstyl et al., 2020) and has been related to features of cortical function, like cortical oscillations and cortical hierarchies (Mahjoory et al., 2020).

## Methods and Stepwise Procedures

### General Principles of Cortical Type Analysis

The rationale of cortical type analysis is based on the systematic and gradual variation of laminar structure observed across neocortical areas of all cortical lobes and systems. These gradients are observed across all mammalian species investigated so far (Garcia-Cabezas et al., 2019). In this paper we analyze isocortical areas of 6 layers (neocortical areas *sensu stricto*) and periallocortical and proisocortical areas of the mesocortex, that either lack or have rudimentary layer IV (neocortical areas *non-sensu stricto*). The areas of the primary olfactory cortex (piriform cortex and anterior olfactory nucleus) and of the hippocampal formation (dentate gyrus, fields in Ammon’s horn, subiculum, presubiculum, parasubiculum, taenia tecta, indusium griseum, and entorhinal cortex) were considered part of the allocortex (Nieuwenhuys et al., 2008; Puelles et al., 2019) and were not included in the analysis. For definitions and equivalences of cortical sectors according to different authors in the literature see Table 1 in Garcia-Cabezas et al., 2019. It is important to note that areas of different type are distributed along the entire surface of the neocortex in gradients of increasing granularization (development of granular layer IV) and laminar elaboration. Transitions from one cortical type to another are gradual and types are topologically arranged without abrupt jumps or breaks along the gradient. Laminar gradients start at the vicinity of olfactory and hippocampal allocortices. The first stage of differentiation in laminar gradients are periallocortical areas with the simplest laminar structure of all neocortical areas; in the last stage of differentiation are the primary sensory (visual, somesthetic, auditory) areas with the most elaborated laminar structure in the neocortex (Sanides, 1970; Reep, 1984; Pandya et al., 2015; Garcia-Cabezas et al., 2019). Tracing laminar elaboration of neocortical areas along those gradients [e.g., in the prefrontal cortex (Barbas and Pandya, 1989); in the temporal lobe (Pandya and Sanides, 1973); in the insula (Mesulam and Mufson, 1982); in frontal motor areas (Barbas and Pandya, 1987)] is required to identify the most useful laminar features for cortical type analysis. Some of these features include development of layer IV, prominence (denser cellularity and larger neurons) of deep (V–VI) or superficial (II–III) layers, definition of sublayers (e.g., IIIa and IIIb), sharpness of boundaries between layers, and presence of large pyramids in superficial layers (Sanides, 1970; Barbas and Pandya, 1989; Goulas et al., 2018; Garcia-Cabezas et al., 2019).

Deformation due to folding of the cerebral cortex is an important factor to take into account when analyzing the systematic variation of laminar architecture in the neocortex. Deep layers are stretched in the fundus of sulci and appear thinner than in the straight parts of the gyrus (dome and sulcal wall); on the contrary, superficial layers are stretched at the point of turning of the gyrus into the sulcus and layer I appears thinner (von Economo, 1927/2009; Bok, 1959; Hilgetag and Barbas, 2005, 2006). Thus, cortical type analysis is optimally performed on micrographs of the straight parts of gyri, but we also examined cortical areas near the turning points of gyri and at the fundus of sulci, to confirm that, despite the deformations due to cortical folding, cortical type analysis yields the same results in straight and turning parts of cortical areas.

It is also important to realize that most of the cortical areas defined by Brodmann (1909/1999) and von Economo and Koskinas (1925/2008) will have just one cortical type, but others may be in the middle of transitions from one type to another.

### Systematic Analysis of Cortical Type in Nissl Stained Sections of the Human Prefrontal Cortex

We traced trends of laminar differentiation in the human prefrontal cortex to identify laminar features that vary systematically across cortical gradients. Two trends of laminar differentiation have been described in the human prefrontal cortex. The paraolfactory trend starts in areas of the posterior orbitofrontal cortex (pOFC) adjacent to the anterior olfactory nucleus and progresses in the rostral and lateral directions across orbital areas (Beck, 1949; Sanides, 1964; Hof et al., 1995). The parahippocampal trend starts in areas of the anterior cingulate cortex (ACC) adjacent to the precommissural (taenia tecta) and supracommissural (indusium griseum) parts of the hippocampal formation and progresses in the anterior and dorsal directions across medial prefrontal areas (Sanides, 1964; Mackey and Petrides, 2014). Both trends converge in the dorsolateral prefrontal cortex (DLPFC) (Sanides, 1964, 1970).

**FIGURE 2:**
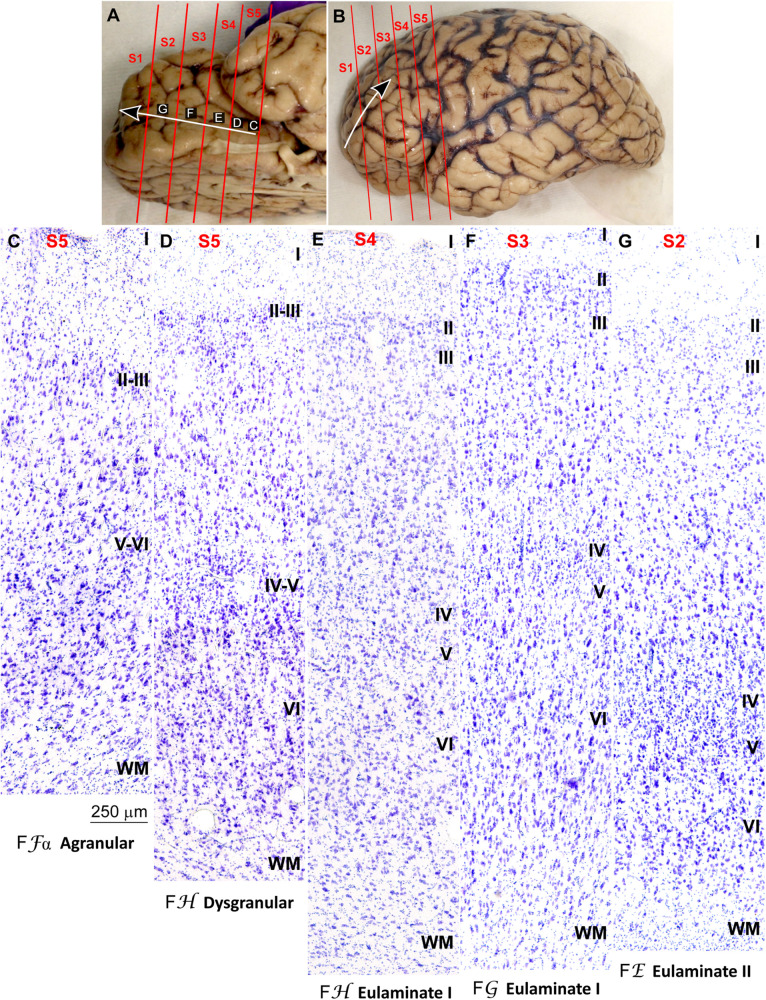
Systematic and gradual variation of laminar structure across areas of the paraolfactory trend in the human prefrontal cortex: Orbitofrontal areas. **(A,B)**, Orbital **(A)** and lateral **(B)** views of the left hemisphere from the brain (case HCD): red lines show the planes of separation of coronal slabs; black and white arrows indicate laminar differentiation along the paraolfactory trend. **(C–G)**, Micrographs of the orbital cortex in Nissl stained sections at the levels indicated in **(A)**; the areas according to the Atlas of von Economo and Koskinas (1925/2008) and the cortical type are indicated below each micrograph; see the text for description of laminar features. S1, S2, S3, S4, S5, indicate coronal slabs from anterior to posterior; WM, white matter. Roman numerals indicate cortical layers. Calibration bar in **(C)** applies to **(C–G)**.

**FIGURE 3:**
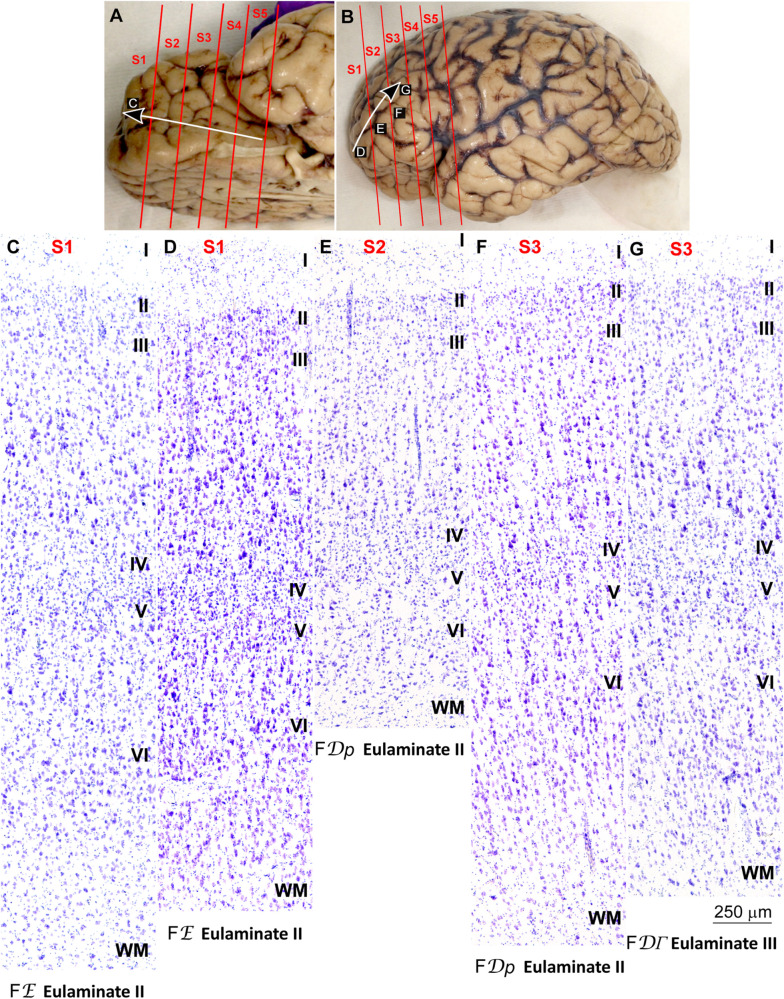
Systematic and gradual variation of laminar structure across areas of the paraolfactory trend in the human prefrontal cortex: Frontopolar and dorsolateral prefrontal areas. **(A,B)**, Orbital **(A)** and lateral **(B)** views of the left hemisphere from the brain (case HCD): red lines show the planes of separation of coronal slabs; black and white arrows indicate laminar differentiation along the paraolfactory trend. **(C–G)**, Micrographs of the frontopolar and dorsolateral prefrontal cortex in Nissl stained sections at the levels indicated in **(B)**; the areas according to the Atlas of von Economo and Koskinas (1925/2008) and the cortical type are indicated below each micrograph; see the text for description of laminar features. S1, S2, S3, S4, S5, indicate coronal slabs from anterior to posterior; WM, white matter. Roman numerals indicate cortical layers. Calibration bar in **(G)** applies to **(C–G)**.

**FIGURE 4:**
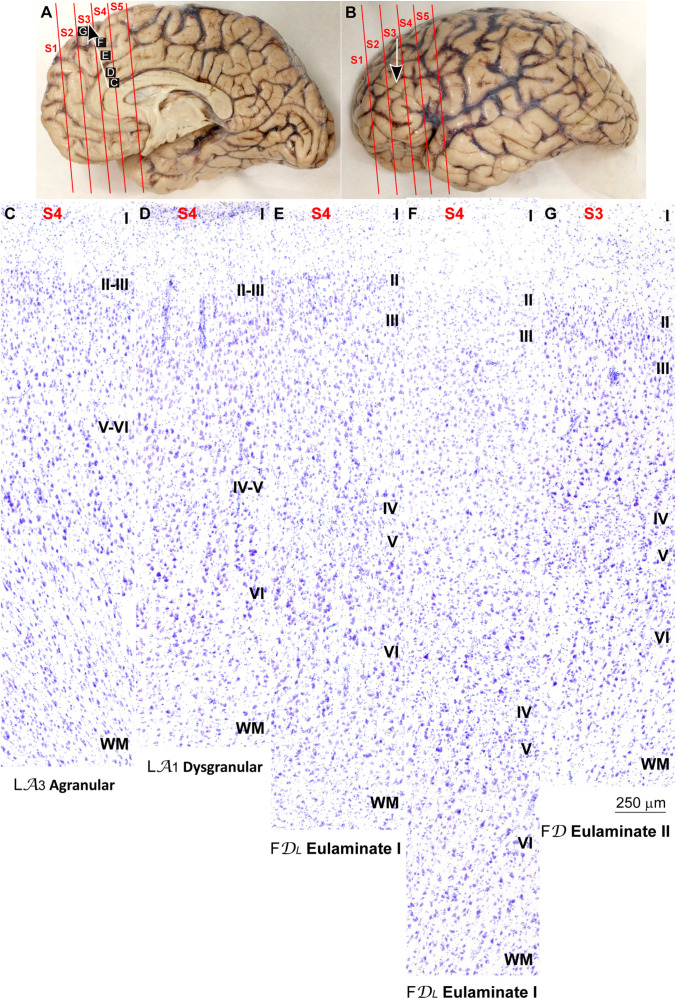
Systematic and gradual variation of laminar structure across areas of the parahippocampal trend in the human prefrontal cortex: Anterior cingulate and medial prefrontal areas. **(A,B)**, Medial **(A)** and lateral **(B)** views of the left hemisphere from the brain (case HCD): red lines show the planes of separation of coronal slabs; black and white arrows indicate laminar differentiation along the parahippocampal trend. **(C–G)**, Micrographs of the anterior cingulate and medial prefrontal cortex in Nissl stained sections at the levels indicated in **(A)**; the areas according to the Atlas of von Economo and Koskinas (1925/2008) and the cortical type are indicated below each micrograph; see the text for description of laminar features. S1, S2, S3, S4, S5, indicate coronal slabs from anterior to posterior; WM, white matter. Roman numerals indicate cortical layers. Calibration bar in **(G)** applies to **(C–G)**.

**FIGURE 5:**
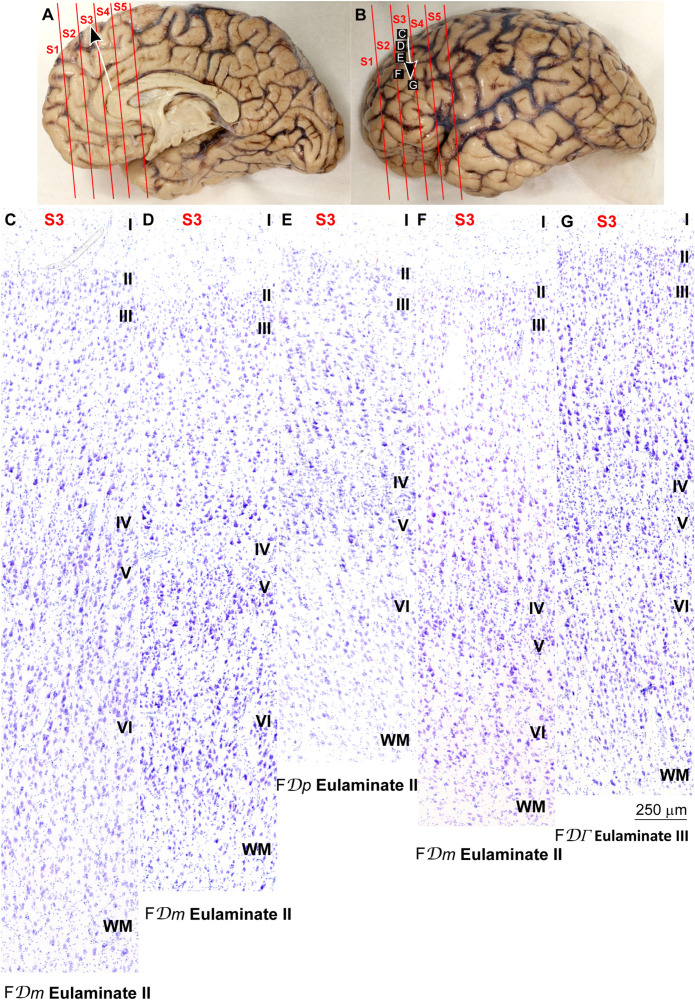
Systematic and gradual variation of laminar structure across areas of the parahippocampal trend in the human prefrontal cortex: Dorsolateral prefrontal areas. **(A,B)**, Medial **(A)** and lateral **(B)** views of the left hemisphere from the brain (case HCD): red lines show the planes of separation of coronal slabs; black and white arrows indicate laminar differentiation along the parahippocampal trend. **(C–G)** Micrographs of the dorsolateral prefrontal cortex in Nissl stained sections at the levels indicated in **(B)**; the areas according to the Atlas of von Economo and Koskinas (1925/2008) and the cortical type are indicated below each micrograph; see the text for description of laminar features. S1, S2, S3, S4, S5, indicate coronal slabs from anterior to posterior; WM, white matter. Roman numerals indicate cortical layers. Calibration bar in **(G)** applies to **(C–G)**.

We traced the paraolfactory trend from pOFC areas across the orbital cortex and the frontal pole up to the most central part of the DLPFC (arrow in Figures 2A,B, 3A,B). We also traced the parahippocampal trend from the ACC across the medial prefrontal cortex (mPFC) up to the DLPFC (arrow in Figures 4A,B, 5A,B). Both trends were traced to a common end point of laminar differentiation (Sanides, 1964, 1970).

The areas in the pOFC adjacent to the anterior olfactory nucleus and the areas in the ACC adjacent to the precommissural and supracommissural parts of the hippocampal formation lacked layer IV and had overall poor laminar differentiation. Deep layers (V–VI) were more prominent than superficial layers (II–III) with more neurons densely packed. Superficial layers were sparsely populated. The boundary between layers I and II was irregular and the largest pyramidal neurons were in layer V (Figures 2C, 4C). We categorized these areas as ***Agranular*** areas.

More anterior in the pOFC and the ACC, cortical areas had rudimentary layer IV with deep layers (V–VI) slightly more prominent than superficial layers (II–III). The boundary between layers I and II was slightly irregular and the largest pyramidal neurons were in layer V (Figures 2D, 4D). We categorized these areas as***Dysgranular***.

Anterior to the pOFC and dorsal to the ACC cortical areas had thin but continuous and regular layer IV. Deep layers V–VI were as prominent as superficial layers II–III, and large pyramids had comparable size in layers III and V. Layers V and VI showed better differentiation than in Agranular and Dysgranular areas and the border between layers I and II was sharper (Figures 2E,F, 4E,F). We categorized these areas as***Eulaminate I***.

Areas in the frontal pole and most areas in the DLPFC had thicker layer IV than Eulaminate I areas. In these areas, superficial layers II–III were more prominent than deep layers V–VI and the largest pyramids were in layers III. Layers V and VI showed sharp differentiation with sublayers and the border between layers I and II was also sharp (Figures 2G, 3C–F, 4G, 5C–F). We categorized these areas as***Eulaminate II***.

Areas in the DLPFC at the end of the parahippocampal and paraolfactory trends had laminar structure comparable to Eulaminate II areas, but the largest pyramids in layer III were larger and more prominent than those seen in Eulaminate II areas (Figures 3G, 5G). We categorized these areas as***Eulaminate III***.

It is important to note that Agranular areas were adjacent to the precommissural and supracommissural parts of the hippocampal formation and the anterior olfactory nucleus of the primary olfactory cortex. More anterior, areas adjacent to Agranular areas were Dysgranular. Then, laminar gradients of differentiation progressed from Dysgranular areas into Eulaminate areas along both trends as described in classical studies (Sanides, 1964). We did not find topological violations along the paraolfactory and parahippocampal gradients of laminar differentiation, which means that transitions form one type to another along those trends always showed an increment or decrement of one. Thus, there were transitions form Dysgranular to Agranular or to Eulaminate I areas, but never from Agranular to Eulaminate I. There were also transitions from Eulaminate II to Eulaminate I or to Eulaminate III, but Eulaminate III areas in the DLPFC were surrounded exclusively by Eulaminate II areas.

In summary, we concluded that the most useful laminar features for cortical type analysis were development of layer IV, relative prominence of superficial (layers II–III) over deep (layers V–VI) laminar groups, laminar distribution of the largest pyramids, definition of sublayers in deep (V–VI) layers, and definition of the boundary between layers I and II. The progressive enlargement of pyramidal neurons in layer III across cortical gradients of laminar differentiation was named externopyramidization (Sanides, 1970; Goulas et al., 2018). This phenomenon is more accentuated in primates compared to rodents and reaches its maximum in Eulaminate areas of the human cortex (García-Cabezas et al., 2018). The variation of laminar features and the definition of cortical types are summarized in Table 2.

**TABLE 2:** Laminar features of cortical types across human neocortex.

**Laminar features**	**Agranular**	**Dysgranular**	**Eulaminate I**	**Eulaminate II**	**Eulaminate III**	**Koniocortex**
Layer IV	Absent	Thin, irregular, discontinuous	Thin, regular, continuous	Thick, regular, continuous	Thick, regular, continuous	Thickest
More prominent laminar group	Deep V-VI	Deep V-VI	Deep V–VI and superficial II–III equally prominent	Superficial II–III	Superficial II–III	Superficial II–III (denser with small neurons)
Largest pyramids	V	V	V and III	III	III (larger)	III
Layers V–VI	Poorly differentiated	Poorly differentiated Well differentiated in cingulate	Well differentiated Some with sublayers	Sublayers	Sublayers	Sublayers
Layers I–II boundary	Irregular	Slightly irregular	Sharp	Sharp	Sharp	Sharp

### Relation Between Cortical Type and SMI-32 Staining

The antibody SMI-32 labels a subset of pyramidal projection neurons in layers III and V and, to a lesser extent, in layers II and VI in the cerebral cortex of primates (Campbell and Morrison, 1989). The density of neurons positive for SMI-32 varies across cortical areas in parallel to laminar elaboration (Hof et al., 1995; Barbas and García-Cabezas, 2015, 2016; García-Cabezas and Barbas, 2017) and thus can be used as a sensitive architectonic marker.

**FIGURE 6:**
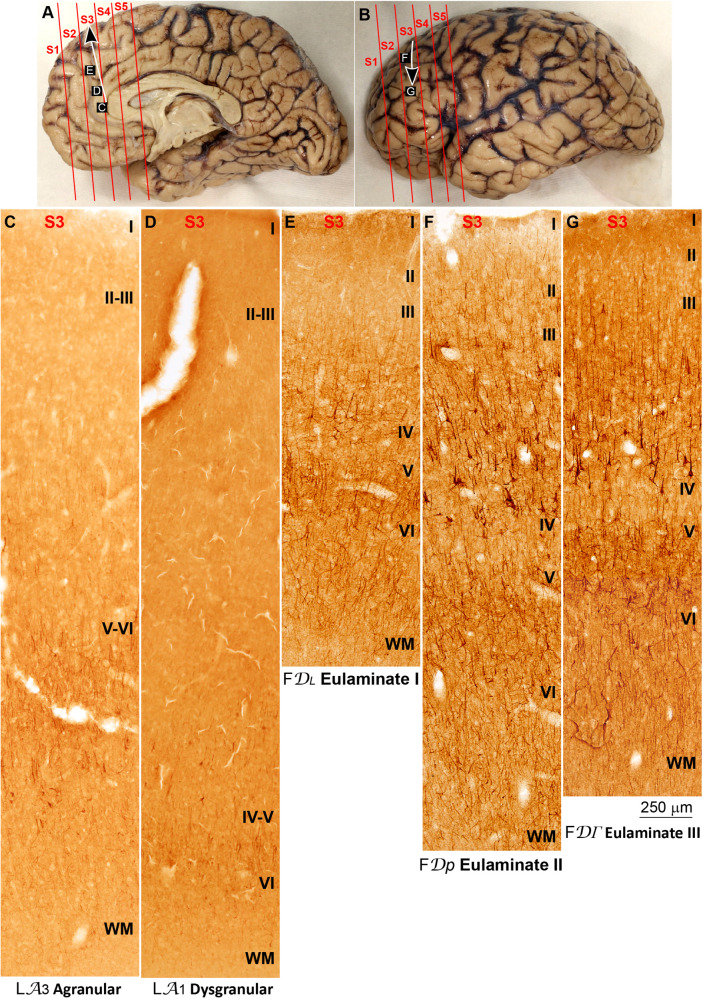
Systematic and gradual variation of laminar structure in SMI-32 immunostained sections across areas of the parahippocampal trend in the human prefrontal cortex. **(A,B)** Medial **(A)** and lateral **(B)** views of the left hemisphere from the brain (case HCD): red lines show the planes of separation of coronal slabs; black and white arrows indicate laminar differentiation along the parahippocampal trend. **(C–G)**, Micrographs of the anterior cingulate, medial prefrontal, and dorsolateral prefrontal cortices in SMI-32 stained sections at the levels indicated in **(A,B)**; the areas according to the Atlas of von Economo and Koskinas (1925/2008) and the cortical type are indicated below each micrograph; see the text for description of laminar features. S1, S2, S3, S4, S5, indicate coronal slabs from anterior to posterior; WM: white matter. Roman numerals indicate cortical layers. Calibration bar in **(G)** applies to **(C–G)**.

We traced variation in SMI-32 immunostaining along the parahippocampal trend (arrow in Figures 6A,B). Agranular areas in the ACC adjacent to the precommissural and supracommissural parts of the hippocampal formation had most labeled neurons for SMI-32 in layers V-VI and few labeled neurons in layer III (Figure 6C). In Dysgranular areas in the ACC there were labeled neurons for SMI-32 in layers V-VI and some in layer III (Figure 6D). Eulaminate I areas in the mPFC had labeled neurons for SMI-32 in layers V–VI and III, delineating a band of unstained tissue corresponding to layer IV (Figure 6E). Eulaminate II areas of the DLPFC had labeled neurons for SMI-32 in layers II–III and V–VI leaving layer IV unstained; some pyramidal neurons in layer III were intensely labeled for SMI-32 (Figure 6F). Labeling for SMI-32 in Eulaminate III areas of the DLPFC was comparable to labeling in Eulaminate II areas but with slightly more densely labeled pyramidal neurons in layer III (Figure 6G).

### Analysis of Cortical Type in Nissl Stained Sections of Human Koniocortical and Parakoniocortical Areas in the Occipital Lobe

We also traced laminar changes across visual areas in the occipital lobe to explore koniocortical and parakoniocortical areas, because these areas have the most elaborated laminar architecture across the entire cortex (Sanides, 1970). Trends of laminar differentiation across occipital visual areas have been described in the human (Vitzthum and Sanides, 1966) and non-human primate (Hilgetag et al., 2016). We traced laminar changes from the primary visual area (Brodmann’s area 17; von Economo and Koskinas area OC) into the adjacent secondary visual area (Brodmann’s area 18; von Economo and Koskinas area OB); we also studied the laminar architecture of area prostriata, the visual area of the occipital lobe with the simplest laminar architecture (Vitzthum and Sanides, 1966).

Before proceeding with the description of laminar features in area 17 we must make some observations on the nomenclature of layers in this area. The most widely used laminar nomenclature labels three sublayers for layer IV: IVa, IVb, and IVc (Brodmann, 1909/1999). Another nomenclature labeled layers IVa and IVb as parts of layer III (Hassler, 1966). Tract-tracing studies in rhesus macaques labeled projection neurons in layers IVa and IVb (Rockland and Pandya, 1979) supporting the notion that these sublayers are part of layer III. Thus, for the analysis of laminar architecture of area 17 we will consider that Brodmann’s layer IVc is layer IV proper and we will consider layers IVa and IVb as parts of layer III.

**FIGURE 7:**
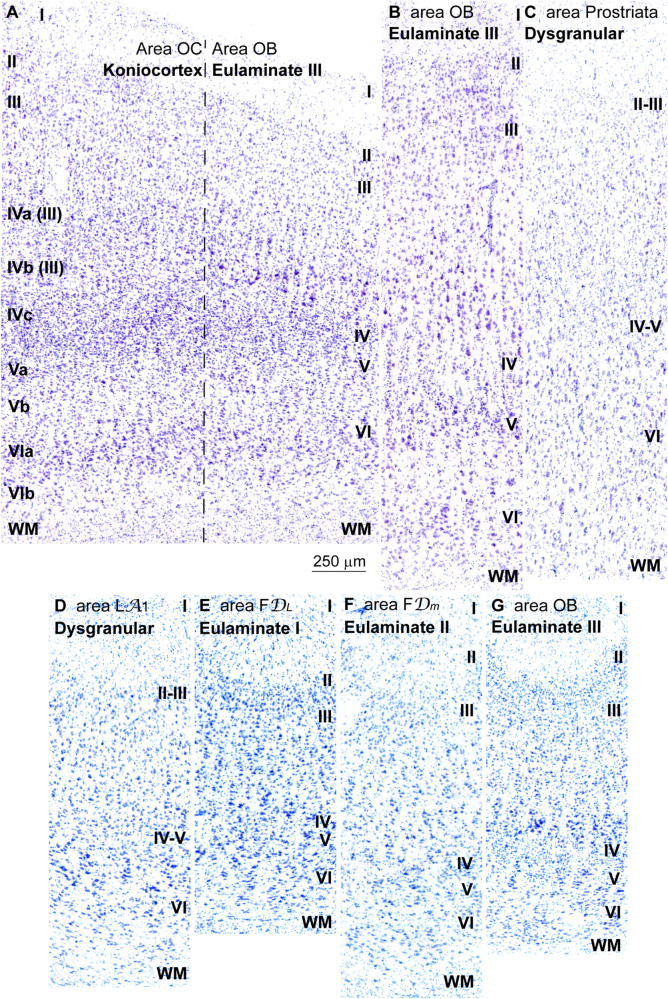
Laminar variation of koniocortical and parakoniocortical areas in the occipital lobe in Nissl stained sections and in sulcal parts of prefrontal and occipital areas. **(A)**, Micrograph of the transition between the primary visual area (area OC, left) and the secondary visual area (area OB, right) in Nissl stained section; roman numerals on the left indicate layers of area OC; roman numerals on the right indicate layers of area OB. It must be considered that the most widely used laminar nomenclature for area OC labels the deepest part of layer III as layers IVa and IVb [note that in the laminar nomenclature of Brodmann (1909/1999) parts of layer III are labeled as IVa and IVb (Hassler, 1966)] only layer IVc is comparable to layer IV in other areas; layers IVa and IVb are part of layer III and contain projection neurons. **(B)**, Micrograph of area OB in a remote sector from area OC in Nissl stained section. **(C)**, Micrograph of area prostriata in Nissl stained section. **(D–G)**, Micrographs of the sulcal parts (fundus) of Dysgranular area LA_1_ **(D)**, Eulaminate I area FD*_*L*_* **(E)**, Eulaminate II area FD*_*m*_* **(F)**, and Eulaminate III area OB **(G)**. The areas according to the Atlas of von Economo and Koskinas (1925/2008) and the cortical type are indicated in each micrograph; see the text for description of laminar features. WM, white matter. Roman numerals indicate cortical layers. Calibration bar in **(A)** applies to **(A–G)**.

Layer IV (layer IVc) of area 17 was thicker than in all the cortical areas studied in the prefrontal cortex. Superficial layers II–III (including layers IVa and IVb) were densely populated with small neurons and deep layers V–VI were sharply subdivided. Pyramidal neurons in layers III and V were not prominent (Figure 7A). The cytoarchitectonic features most characteristic of area 17 were the thicker and denser layer IV and the high density of neurons in superficial layers. We categorized this area as***Koniocortex***, which means granular cortex (von Economo and Koskinas, 1925/2008; von Economo, 1927/2009).

The transition from koniocortical area 17 to parakoniocortical area 18 was sharp (dashed line; Figure 7A). Laminar features of area 18 were comparable to those described in Eulaminate III areas of the DLPFC (Figures 7A,B). On the other hand, area prostriata (Figure 7C), a visual area in the occipital lobe adjacent to the hippocampal formation (Ding et al., 2016), had the simplest laminar architecture comparable to Agranular and Dysgranular areas in the pOFC (Figures 2C,D) and the ACC (Figures 4C,E).

We performed cortical type analysis in Nissl stained sections in straight parts (dome and sulcal wall) and in convex and concave turning gyral (dome to sulcal wall) and sulcal (in the fundus) regions of prefrontal and occipital visual areas. The variation of laminar features and the definition of cortical types summarized in Table 2 applies both to straight parts, turning points and sulcal fundus of cortical areas. It should be noted that deformation of the cortical mantle due to folding affects the thickness of cortical layers, but the key features of cortical type analysis like prominence of layers II–III and V–VI, presence and development of layer IV, laminar distribution of the largest pyramids, and definition of layers and sublayers do not change and allow proper analysis of cortical type in straight parts, turning points and sulcal fundi of cortical areas (Figures 7D–G).

### Systematic Analysis of Cortical Type in the Atlas of the Human Cerebral Cortex of von Economo and Koskinas (1925/2008)

We applied the criteria summarized in Table 2 for categorizing cortical types in human prefrontal areas and visual areas in the occipital lobe described above to the micrographs of the Atlas of the Human Cerebral Cortex of von Economo and Koskinas (1925/2008). This Atlas surveys more than 90 neocortical areas across all cortical lobes and systems and is illustrated with high quality micrographs. The micrographs of the Atlas of von Economo and Koskinas (1925/2008) were taken from sections cut perpendicular to the long axis of straight parts of each area, either at the dome of the gyrus or at the gyral wall in the sulcus. Therefore, in some areas the plane of sectioning in the Atlas of von Economo and Koskinas (1925/2008) did not coincide with the coronal plane. The cortical type analysis described above was performed in coronal sections through prefrontal and occipital visual areas. Careful side-by-side examination of micrographs from the Atlas and micrographs of our coronal sections from matching areas showed that, when the plane of sectioning was not matched in the two datasets, the relative thickness of the layers was different, but applying the laminar features summarized in Table 2 to both micrographs resulted in the definition of the same cortical type.

We first examined blindly scans of the micrographs depicted in the Atlas and applied the criteria summarized in Table 2 for categorizing cortical areas as ***Agranular***, ***Dysgranular***, ***Eulaminate I***, ***Eulaminate II***, ***Eulaminate III***, or ***Koniocortical***. Then, we repeated the analysis unblinded to check for topological violations in cortical type grading.

During the analysis of micrographs of neocortical areas from the Atlas of von Economo and Koskinas (1925/2008) we found that some areas had laminar features of Eulaminate areas, like larger pyramids in layer III, but seemed to lack layer IV and both deep (V–VI) and superficial (II–III) layers were equally prominent. These areas were characterized by lower gray/cell index [surface of unstained neuropil/surface of stained cells in Nissl sections examined microscopically (von Economo and Koskinas, 1925/2008; Haug, 1956)], with more unstained neuropil between neuron bodies and glial cell nuclei, sharp boundary between layers I and II, and absence of sublayer differentiation in deep layers V and VI. A subsequent unblinded analysis showed that these areas were premotor and primary motor areas in the frontal lobe. Traditionally it was considered that frontal motor areas lacked layer IV and were termed Agranular together with periallocortical Agranular areas (e.g., Parent, 1996; Amaral, 2000), but premotor areas and the primary motor cortex in adult primates do have layer IV as shown by SMI-32 staining and quantitative stereological methods (García-Cabezas and Barbas, 2014). Actually, layer IV is marked with other layers in the margins of micrographs of frontal motor areas in the Atlas of von Economo and Koskinas (1925/2008).

### Systematic Analysis of Cortical Type in Premotor and Primary Motor Areas

The unblinded analysis also showed that motor areas in the human frontal lobe, like neocortical areas in sensory systems, are arranged along gradients of laminar differentiation as described in the macaque cortex (Barbas and Pandya, 1987; Morecraft et al., 2012). ***Agranular motor*** and ***Dysgranular motor*** areas had comparable laminar architecture to other limbic areas, as described for the prefrontal cortex, but some motor Agranular and Dysgranular areas in the cingulate cortex had well differentiated layers V and VI. These areas, like Agranular and Dysgranular areas in the insula, were enriched in von Economo neurons. ***Eulaminate I premotor*** areas had large pyramids in layers III and V forming a band in the middle part of these areas. The next level in pre-motor areas had larger pyramids in layer III although few Betz cells, which were larger, could be in layer V (***Eulaminate II premotor***). Finally, the ***Primary motor*** area at the end of the laminar trend had larger pyramids in layer III than in V with the exception of Betz cells. We considered the primary motor cortex as ***Eulaminate III***. The specific laminar features for cortical type analysis of frontal motor areas are summarized in Table 3.

**TABLE 3:** Laminar features of cortical types in motor areas of the human frontal cortex.

**Laminar features**	**Agranular motor**	**Dysgranular motor**	**Eulaminate I (Premotor)**	**Eulaminate II (Premotor)**	**Eulaminate III (Primary motor)**
Layer IV	Absent	Thin, irregular, discontinuous	Non-visible with Nissl stain	Non-visible with Nissl stain	Non-visible with Nissl stain
Gray/cell index	Low	Low	High	High	High
More prominent laminar group	Deep V–VI	Deep V–VI	Deep V–VI and superficial II–III	Deep V–VI and superficial II–III	Deep V–VI and superficial II–III
Largest pyramids	V	V	V and III	III	III Betz cells in V
Layers V–VI	Poorly differentiated	Poorly differentiated	Well differentiated	Well differentiated	Well differentiated
Layers I–II boundary	Irregular	Slightly irregular	Sharp	Sharp	Sharp

## Anticipated Results

### Summary Tables for Cortical Types in the Atlas of the Human Cerebral Cortex of von Economo and Koskinas (1925/2008)

We summarized the cortical type analysis of neocortical areas of the Atlas of von Economo and Koskinas (1925/2008) in Table 4 (Frontal lobe, premotor and primary motor), Table 5 (Frontal lobe, prefrontal), Table 6 (Cingulate gyrus, Insula, and Temporal Lobe), and Table 7 (Parietal and Occipital lobes). These authors used a symbolic notation to label cortical areas. Each symbol comprises a Roman capital letter from the initial of the respective lobe, a calligraphic capital for the sequence of a gyrus within a lobe, and a Latin or Greek subscript for the microscopic features of the area (Triarhou, 2007). In contrast, Brodmann used a numerical system to label the areas in his maps of the cerebral cortex. Brodmann described 43 areas in the human cerebral cortex and 30 areas in the cortex of apes (Brodmann, 1909/1999). Each Brodmann’s area of the human cortex is labeled with a number between 1 and 52, but areas with the numbers 12–16 and 48–51 are not shown in Brodmann’s map (Zilles and Amunts, 2010).

**TABLE 4:** Areas in the human cerebral cortex according to von Economo and Koskinas (1925/2008) and their corresponding types: Frontal lobe, premotor and primary motor areas.

**Cortical region**	**Abbreviation**	**Cortical area**	**Plate number**	**Cortical type**
Frontal lobe (F)	F𝒜	Precentral area	−	Eu-III**
Motor and	F𝒜_γ_	Giant pyramidal precentral area	1, 2, 3, 4, 66	Eu-III
premotor areas	F𝒜_*op*_	Precentral area in operculum	−	Eu-III**
	F𝒜ℬ	*Transitional area*	5	Eu-II
	Fℬ	Agranular frontal area	6	Eu-II
			7, 8	Eu-I
	Fℬ_*op*_	Agranular frontal area in operculum	10	Eu-I
	Fℬ(𝒞)	*Transitional area*	9	Eu-II
	Fℬ(𝒞)_*op*_	*Transitional area*	10	Eu-I
	Fℬ𝒞	*Transitional area*	11	Eu-I
	F𝒞	Intermediate frontal area	12, 13	Eu-I
	F𝒞**L**	Intermedio-limbic frontal area	17	Dys
	F𝒞ℬ*_*m*_*	Broca’s area (magnocellular agranular intermediate frontal area)	14, 15	Eu-II
	F𝒞**I**	Intermediate frontal area at the beginning of the insula	−	Eu-I**

**Cortical type classifications were derived based on neighboring classifications and topological organization of cortical types along gradients of laminar differentiation, as no picture was available in the Atlas of von Economo and Koskinas (1925/2008).

**TABLE 5:** Areas in the human cerebral cortex according to von Economo and Koskinas (1925/2008) and their corresponding types: Frontal lobe, prefrontal areas.

**Cortical region**	**Abbreviation**	**Cortical area**	**Plate number**	**Cortical type**
Frontal lobe (F) Prefrontal areas	F𝒞_*op*_	Opercular intermediate frontal area	16	Eu-I
	F𝒞(𝒟)_op_	*Transitional area*	16	Eu-I
	F𝒞(𝒟)	*Transitional area*	18	Eu-I
	F𝒟𝒞	*Transitional area*	20	Eu-II
	F𝒟	Granular frontal area	24	Eu-II
	F𝒟*_*m*_*	Magnocellular granular frontal area	19	Eu-II
			21	Eu-I
			23	Dys
	F𝒟*_*m*_ (𝒞)*	*Transitional area*	19	Eu-II
	F𝒟*_*m*_* (ℰ)	*Transitional area*	21	Eu-I
	F𝒟*_*m*_/*F𝒟*_*p*_*	*Transitional area*	23	Dys
	F𝒟*_*p*_*	Parvicellular granular frontal area	22	Eu-II
			23	Dys
	F𝒟_*op*_	Granular frontal area in operculum	16	Eu-I
	F𝒟ℒ	Limbic granular frontal area	26	Eu-I
	F𝒟*Δ*	Middle granular frontal area	27	Eu-II
	F𝒟*Γ*	Triangular frontal area	28–30	Eu-III
	Fℰ	Frontopolar area	31	Eu-II
	Fℰℒ	Limbic frontopolar area	−	Eu-I
	Fℱ_*g*_	Granular orbital area	32	Eu-I
	Fℱ_α_	Agranular orbital area	33	Dys
	Fℱ_φ_	Pretriangular orbital area	34	Eu-I
	F𝒢	Area of straight gyrus (area recta)	35	Eu-I
	F𝒢*_*i*_*	Internal straight area	−	Eu-I**
	Fℋ	Prefrontal area	37	Eu-I
	Fℋℒ’	Paraolfactory prefrontal area	39	Dys
	Fℋ**L**	Limbic prefrontal area	38	Dys
	F𝒿	Frontoinsular area	42	Ag
			44	Dys
	F𝒦	Frontal piriform area	−	Dys-Ag**
	Fℒ_1_	Primary paraolfactory area	39, 40	Dys
	Fℒ_2_	Secondary paraolfactory area	40	Ag
	Fℒ_3_	Tertiary paraolfactory area	40	Ag
	Fℳ	Geniculate area	40	Ag
	Fℳ*_*t*_*	Geniculate area of olfactory triangle	−	Ag**

**Cortical type classifications were derived based on neighboring classifications and topological organization of cortical types along gradients of laminar differentiation, as no picture was available in the Atlas of von Economo and Koskinas (1925/2008).

**TABLE 6:** Areas in the human cerebral cortex according to von Economo and Koskinas (1925/2008) and their corresponding types: Limbic, insular, and temporal lobes.

**Cortical region**	**Abbreviation**	**Cortical area**	**Plate number**	**Cortical type**
Superior limbic lobe (L) areas	L𝒜_1_	Precingulate agranular anterior limbic area	45	Ag
	L𝒜_2_	Anterior cingulate agranular anterior limbic area	46	Ag
	L𝒜_3_	Cingulate agranular anterior limbic area limitans	47	Dys-Ag
	L𝒞_1_	Dorsal posterior cingulate area	48	Eu-I
	L𝒞_2_	Ventra posterior cingulate area	49	Dys
	L𝒞_3_	Posterior cingulate area limitans	50	Ag
	L𝒟	Agranular retrosplenial area	52	Ag
	Lℰ_1_	Superior retrosplenial area granulosa	52	Dys
	Lℰ_2_	Inferior retrosplenial area granulosa	52	Ag
Insular lobe (I) areas	I𝒜_1_	Dorsal precentral insular area	53	Dys
	I𝒜_2_	Ventral precentral insular area	54, 55	Dys
	I𝒜_2_ (B)	*Transitional area*	55	Eu-I
	Iℬ	Post-central insular area	55, 56	Eu-I
	Iℬ𝒯	Post-central insular area at temporal entrance	57	Eu-I
	I𝒞	Orbito-insular area	58	Dys-Ag
	I𝒟	Piriform insular area	58	Dys-Ag**
Temporal lobe (T) areas	T𝒜_1_	Posterior superior temporal area	88, 89	Eu-II
			92	Eu-III
	T𝒜_2_	Anterior superior temporal area	−	Eu-I**
	Tℬ	Magnocellular supratemporal area simplex	93	Eu-III
	T𝒞	Supratemporal area granulosa	94	Koniocortex
	T𝒟	Intercalated supratemporal area	95	Koniocortex
	Tℰ_1_	Middle temporal area proper	89, 90	Eu-III
	Tℰ_2_	Inferior temporal area proper	91	Eu-I
	Tℱ	Fusiform area	96	Eu-I
	Tℋ	Hippocampotemporal area	108	Ag
	Tℋα	Agranular hippocampotemporal area	−	Ag**
	T𝒢	Temporopolar area	97	Dys
	T𝒢α	Agranular temporopolar area	98, 99	Ag
	Tℐ	Temporal piriform area	98	Ag
	T𝒦	Posterior area of substantia perforate	−	Dys-Ag**

**Cortical type classifications were derived based on neighboring classifications and topological organization of cortical types along gradients of laminar differentiation, as no picture was available in the Atlas of von Economo and Koskinas (1925/2008).

**TABLE 7:** Areas in the human cerebral cortex according to von Economo and Koskinas (1925/2008) and their corresponding types: Parietal and occipital lobes.

**Cortical region**	**Abbreviation**	**Cortical area**	**Plate number**	**Cortical type**
Parietal lobe (P) areas	P𝒜_1_	Giant pyramidal post-central area	59	Eu-II
	P𝒜_2_	Giant pyramidal post-paracentral area	59, 66	Eu-II
			67	Eu-III
	Pℬ_2_	Oral post-central area simplex	60, 61	Koniocortex
	Pℬ_1_	Oral post-central area granulosa	59, 60, 61, 62	Koniocortex
	P𝒞	Intermediate post-central area	63	Eu-III
	P𝒞γ	*Transitional area*	64	Eu-III
	P𝒟	Caudal post-central area	65	Eu-II
	P(𝒟)ℰ	Superior parietal area (transition parietal post-central area)	65, 66	Eu-II
	Pℰ*_*m*_*	Magnocellular superior parietal area	68, 69	Eu-III
	Pℰ*_*p*_*	Parvicellular superior parietal area	70	Eu-II
	Pℰγ	Giant pyramidal posterior superior parietal area	71	Eu-III
	Pℱ	Supramarginal area	72, 73	Eu-II
			74	Eu-I
	Pℱ*_*t*_*	Tenuicortical supramarginal area	75	Eu-I
	Pℱ_*op*_	Opercular supramarginal area	77	Eu-III
	Pℱ*_*cm*_*	(posterior) magnocellular supramarginal area	75	Eu-I
	P𝒢	Angular area	76	Eu-I
	Pℋ𝒫	Basal (temporooccipital) parietal area at parietal entrance	78	Eu-II
	Pℋ𝒯	Basal (temporooccipital) parietal area at temporal entrance	80	Eu-I
	Pℋ𝒪	Basal (temporooccipital) parietal area at occipital entrance	79	Eu-III
Occipital lobe (O) areas	O𝒜_2_	Anterior peristriate area	81	Eu-III
	O𝒜_1_	Posterior peristriate area	82, 83	Eu-III
	O𝒜*_*m*_*	Magnocellular peristriate area	81	Eu-III
	Oℬ	Parastriate area	83, 84, 85, 87	Eu-III
	O𝒜γ	Giant pyramidal parastriate boundary	85, 86, 87	Koniocortex
	Oℬ_Ω_	Maculae granulosae of parastriate area	−	Eu-III**
	O𝒞	Striate area (granulosa)	85, 86, 87, 88	Koniocortex

**Cortical type classifications were derived based on neighboring classifications and topological organization of cortical types along gradients of laminar differentiation, as no picture was available in the Atlas of von Economo and Koskinas (1925/2008).

In Tables 4–7, we indicate the symbol of each cortical area according to von Economo and Koskinas (1925/2008), the number of the plates of their Atlas in which cortical type analysis was performed, and the corresponding cortical type.

### Maps of the Distribution of Cortical Types in the Atlas of the Human Cerebral Cortex of von Economo and Koskinas (1925/2008)

**FIGURE 8:**
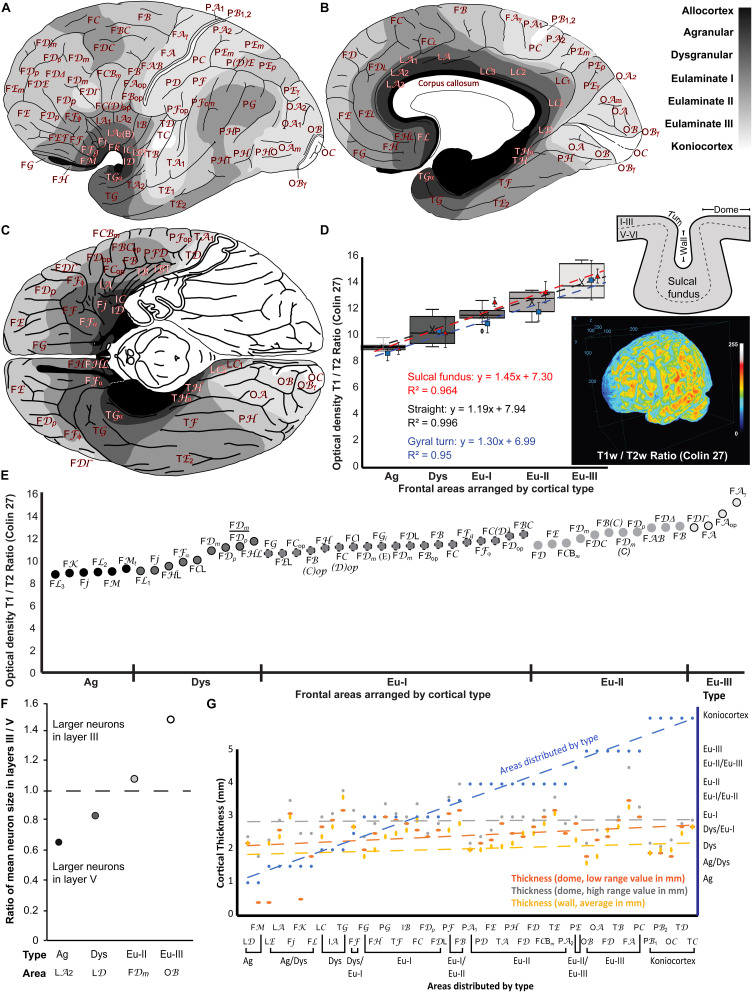
Cortical types across the maps of the human cerebral cortex of the Atlas of von Economo and Koskinas (1925/2008) and comparison of cortical type maps with T1w/T2w MRI maps, size of pyramidal neurons in layers III and V, and cortical thickness. **(A)**, Lateral view of the hemisphere. **(B)**, Medial view of the hemisphere. **(C)**, Basal view of the brain; the temporal pole has been removed on the left hemisphere. In **(A–C)** frontal is to the left and occipital to the right. Cortical types are depicted in grayscale according to the analysis summarized in Tables 4–7. The allocortex is colored black, Agranular areas are colored with the darkest gray, Dysgranular and Eulaminate areas are colored in progressively lighter grays and Konicortices are white. Cortical areas are indicated with the alphanumeric nomenclature of von Economo and Koskinas (1925/2008). **(D)**, Box plots of the mean optical density estimates measured at the straight parts (dome and gyral wall) of each cortical area shown in **(E)**. Estimates for each type include the Minimum (smallest value in a data set), First quartile (middle value between the Minimum and Median—25th percentile), Median (middle value of a data set), Third quartile (middle value between the Median and the Maximum—75th percentile), and Maximum (largest value in a data set). The black dotted line indicates the linear fit (*R*^2^ = 0.996) with a slope = 1.19. We also included measurements and the linear fits at the sulcal fundus (red dashed line), and gyral turning points (blue dashed line) of each cortical area. Three-dimensional projection of the T1w/T2w ratio of the Colin 27 dataset that was used for this analysis. **(E)**, Mean optical density estimates of representative cortical columns of all 47 frontal regions identified in the Atlas of the Human Cerebral Cortex of von Economo and Koskinas (1925/2008), color-coded in grayscale and grouped by respective cortical type, as presented in Tables 4, 5. The T1w/T2w ratio, which correlates to myelin content and cytoarchitecture, increases gradually from Agranular to Eulaminate frontal areas. **(F)**, Ratio of mean pyramidal neuron body size in layers III and V shows progressive increase of the size of neuron bodies of pyramidal neurons in layer III in parallel with laminar elaboration. Pyramids with the largest neuron bodies in granular area LA_2_ and Dysgranular area LD are in layer V; in contrast, pyramids with the largest neuron bodies in Eulaminate II area FD*_*m*_* and Eulaminate III area OB are in layer III. **(G)**, Cortical thickness is not related to cortical type. The thickness of 43 cortical areas at the dome (orange dashed line: low range value; gray dashed line: high range value) and at the gyral wall (yellow dashed line) was obtained from von Economo (1927/2009). The 43 analyzed cortical areas are distributed according to cortical type (blue dashed line; see Tables 4–7). Ag, Agranular; Dys, Dysgranular; Eu-I, Eulaminate I; Eu-II, Eulaminate II; Eu-III, Eulaminate III. For the abbreviations of cortical areas (see Tables 4–7).

We represented cortical types in gray scale on the maps of the human cerebral cortex of von Economo and Koskinas (1925/2008). We colored allocortical areas in black, Agranular areas in the darkest gray, and Dysgranular and Eulaminate areas in progressively lighter grays. Koniocortices were colored white. The space between areas that was not sampled in the Atlas of von Economo and Koskinas (1925/2008) was colored according to topological rules; for example, transitions from Agranular to Eulaminate I areas were considered Dysgranular. Thus, the gray scale in the maps represents progressive laminar elaboration across cortical areas. The maps are shown in Figures 8A–C.

### Comparison of Cortical Types in the Frontal Cortex With T1w/T2w MRI Signal Map

As a proof of concept, we applied the results of systematic analysis of cortical types based on high-resolution histological and microscopic approaches to non-invasive, albeit lower-resolution, MRI scans that are commonly used as reference models and spaces in imaging studies. We specifically used the T1-weighted (T1w) and T2-weighted (T2w) signals from the MRI scan of the Colin 27 average brain dataset that is accessible for public use (stereotaxic registration model, high-resolution version 2008; Copyright (C) 1993–2009 Louis Collins, McConnell Brain Imaging Centre, Montreal Neurological Institute, McGill University). We used Image J to align the scans and then estimated the T1w/T2w ratio using the Ratio Plus plugin. As noted earlier for the histological analysis, we estimated T1w/T2w ratios in straight parts of each area, either at the dome of the gyrus or at the gyral wall in the sulcus, but also in turning regions between the domes and walls of gyri as well as the fundus of sulci to examine the effects of compression and stretching of layers in convex and concave cortical regions. The T1w/T2w ratio has been used successfully to differentiate and map cortical areas before (e.g., Glasser and Van Essen, 2011). This approach has several advantages because pairwise division of aligned T1w by T2w images results in cancelation of the intensity bias field contribution of receiver coils, and increases the contrast related to myelin content (increased contrast-to-noise ratio).

We related the T1w/T2w ratio to cortical types in the frontal lobe (Figures 8D,E). We focused on the frontal lobe because we systematically analyzed prefrontal areas in Nissl-stained sections in our lab and prefrontal, premotor, and primary motor areas from the Atlas of von Economo and Koskinas (1925/2008). In addition, previous studies have successfully correlated structural features of the cortex with connections, mainly relying on frontal cortices, where we have detailed quantitative cytoarchitectonic and connectivity datasets in non-human primates (e.g., Barbas and Rempel-Clower, 1997; Morecraft et al., 2012; Hilgetag et al., 2016; Beul et al., 2017; Zikopoulos et al., 2018). We estimated the optical density of the T1w/T2w ratio from representative cortical columns in the MRI scan of the Colin 27 average brain dataset of all 47 frontal neocortical areas identified in the Atlas of von Economo and Koskinas (1925/2008). We then grouped these frontal areas into their respective cortical types (Agranular, Dysgranular, Eulaminate I, Eulaminate II, and Eulaminate III; Tables 4–6) and plotted the mean T1w/T2w optical density ratio of each type (Figures 8D,E). This analysis showed that the T1w/T2w ratio increased following a linear trend along gradients of laminar differentiation from Agranular and Dysgranular areas to progressively more Eulaminate areas (slope: 1.18, *R*^2^ = 0.99, Mean ± SD Ag: 9.09 ± 0.36; Dys: 10.42 ± 1.06; Eu-I: 11.52 ± 0.55; Eu-II: 12.51 ± 0.76; Eu-III: 13.98 ± 1.4; Figure 8D), validating the use of the T1w/T2w ratio for the reliable identification of cortical areas of comparable laminar architecture. Findings were similar in straight parts of the cortex (dome or wall of gyri) and in the convex and concave turning gyral and sulcal (in the fundus) regions of the cortical areas analyzed (Figure 8D). Moreover, in order to examine the effects of the plane of sectioning, we estimated the T1w/T2w ratio using coronal, sagittal, and horizontal planes of the scans after reslicing. Analysis of these datasets produced comparable results, showing no major effects of the plane of sectioning on the T1w/T2w ratio.

### Comparison of Cortical Types With Pyramidal Neuron Body Size in Layers III and V

We measured the size of pyramidal neuron bodies in layers III and V in scans of micrographs of the Atlas of von Economo and Koskinas (1925/2008) and compared these quantitative data with the subjective estimation of the relative size of pyramidal neuron bodies in layers III and V and the other features of cortical type analysis summarized in Table 2. The largest pyramidal neurons in Agranular and Dysgranular areas were found in layer V. In contrast, pyramidal neurons in layer III increased their size progressively across Eulaminate areas in parallel to laminar elaboration (Sanides, 1970; Goulas et al., 2018; see Table 2).

We measured the area of the largest pyramids in layers III and V from representative areas of different cortical types (Agranular area LA_2_ in a region equivalent to Brodmann’s area 24a, Dysgranular area LD in a region equivalent to area prostriata, Eulaminate II area FD*_*m*_* in a region equivalent to Brodmann’s area 46, and Eulaminate III area OB, which is equivalent to Brodmann’s area 18). We divided the average size of the largest pyramids in layer III by the average size of the largest pyramids in layer V in each analyzed area to obtain a ratio that expresses the laminar distribution of the largest pyramids (ratio = 1 when the largest pyramids have comparable size in layers III and V; ratio < 1 when the largest pyramids are in layer V; and ratio > 1 when the largest pyramids are in layer III; Figure 8F). In Agranular area LA_2_ and in Dysgranular area LD the largest pyramids were found in layer V (ratio < 1); in contrast, in Eulaminate II area FD*_*m*_* and in Eulaminate III area OB the largest pyramids were found in layer III (ratio > 1). The quantitative data summarized in Figure 8F confirms the subjective appreciation of progressive increase in the size of pyramidal neurons in layer III in parallel to laminar elaboration.

### Comparison of Cortical Types With Cortical Thickness

We also analyzed the relation between cortical thickness and cortical type because cortical thickness is frequently measured in imaging studies (e.g., Fischl and Dale, 2000; Scholtens et al., 2015; Wagstyl et al., 2020) and has been related to features of cortical function like cortical oscillations and cortical hierarchies (Mahjoory et al., 2020). Data of cortical thickness measured in 43 cortical areas at the dome (gyral straight part) and at the gyral wall was obtained from von Economo (1927/2009). We compared the data of cortical thickness of these 43 cortical areas with their cortical types summarized in Tables 4–7. In areas with more than one type we computed the average type after the following scoring: Agranular = 1, Dysgranular = 2, Eulaminate I = 3; Eulaminate II = 4; Eulaminate III = 5; Koniocortex = 6. Thus, the average type for area FB [(Eulaminate I + Eulaminate II)/2] was 3.5.

The 43 human cortical areas analyzed were distributed by average type in Figure 8G (blue line). Neither of the three measurements of cortical thickness obtained from von Economo (1927/2009) [dome, low range value (orange line, Figure 8G); dome, high range value (gray line, Figure 8G); wall, average value (yellow line, Figure 8G)] were related to cortical type as shown by comparison of the slopes (general linear regression model).

## Discussion

### Cortical Type Parcellation of the Human Neocortex: Differences Across Studies

In the previous sections of this Protocol Paper we describe the laminar features observed in Nissl stained sections that vary systematically across the human neocortex and how to assess these variations qualitatively to categorize cortical types. This analysis allows the division of the human neocortex in several sectors of different laminar elaboration with invariant topological relations. Thus, cortical types are qualitative, but not arbitrary, divisions along a continuum of laminar changes in the neocortex. We categorized 6 cortical types along this continuum in the human neocortex, but simpler and more complex divisions could be done by other researchers as long as they keep the invariant topological relations between types. For instance, 8 cortical types were defined in visual areas of the macaque cortex (Hilgetag et al., 2016) and others classified all the areas of the human neocortex in 3 types: limbic (Agranular and Dysgranular), Eulaminate, and Koniocortex (Zhang et al., 2020). Besides Nissl staining, immunohistochemistry for SMI-32, a sensitive architectonic marker, can help categorize cortical types in humans [as shown here (Figure 6), and in human (Hof et al., 1995) and in non-human primates (Barbas and García-Cabezas, 2015, 2016; García-Cabezas and Barbas, 2017)]. Quantification of cytoarchitectonic features, like the size of pyramidal neuron bodies in layers III and V, could also help categorizing cortical types (Figure 8F).

The laminar features summarized in Tables 2, 3 will help researchers who study the human cerebral cortex to implement their own analyses of laminar structure in Nissl stained sections (processed in their laboratories or scanned and freely available, e.g., Wagstyl et al., 2020), but they may define 6 cortical types, more than 6, or fewer (e.g., only one type of Eulaminate cortex). Differences in the number of cortical types defined will still allow comparison across studies as long as the topological invariant relations of each type defined are preserved in relation with the other types: areas with the poorest laminar differentiation should always be adjacent to the allocortex and areas with the best laminar differentiation should always be the primary sensory areas.

In other words, cortical type analysis allows for (simple or more exhaustive) qualitative divisions of the continuum of laminar changes along the neocortex as long as the topological invariant relations of each type defined are preserved in relation with the other types.

### Practical Utility of Cortical Types Analysis of the Atlas of the Human Cerebral Cortex of von Economo and Koskinas (1925/2008)

The results of systematic analysis of cortical types across micrographs of the Atlas of von Economo and Koskinas are summarized in Tables 4–7. We performed cortical type analysis (Agranular, Dysgranular, Eulaminate I, Eulaminate II, Eulaminate III, Koniocortex) on each plate in the Atlas encompassing 100 neocortical areas, subareas, and transitional zones between areas across the entire cortical surface (Tables 4–7). The micrographs scanned from the Atlas of von Economo and Koskinas (1925/2008) were taken from sections cut perpendicular to the long axis of straight parts of each area, either at the dome of the gyrus or at the gyral wall in the sulcus. We carefully examined side-by-side the micrographs from the Atlas and the micrographs of our coronal sections from matching areas. This comparison showed that, when the plane of sectioning was not matched in the two datasets, the relative thickness of the layers was different, likely due to mechanical factors (Van Essen, 1997; Hilgetag and Barbas, 2006). The effect of those mechanical factors on laminar thickness did not obscure cortical type analysis. Actually, the laminar features summarized in Table 2 applied to micrographs of the same area from the Atlas of von Economo and Koskinas (1925/2008) and from our coronal sections, yielded the same result.

The classification of human neocortical areas into cortical types presented in this protocol paper will guide future studies of the human cerebral cortex because laminar differentiation correlates with multiple aspects of neocortical structure and function, like connectivity and hierarchical processing. Cortical types summarized in Tables 4–7 will allow predictions of laminar patterns of connections between cortical areas, synaptic plasticity, and selective vulnerability to brain diseases, as we will outline in the following sections.

### Modeling Cortical Connectivity and Hierarchies Using Cortical Types

Cortical type, as defined by the underlying cortical architecture, is related to cortico-cortical connections, as described in a relational model: The Structural Model for Connections (Barbas, 1986, 2015; Barbas and Rempel-Clower, 1997; Garcia-Cabezas et al., 2019), also known as Architectonic Type Principle (Hilgetag et al., 2019). According to the Structural Model, the laminar pattern of connections between two given areas depends on the relationship of the cortical type of those areas. Thus, pathways from areas with poor laminar elaboration to areas with better laminar elaboration originate from deep (V–VI) layers and terminate in superficial (I–III) layers (“feedback” pattern). In the reverse direction—from Eulaminate to Agranular and Dysgranular areas—pathways originate mostly in superficial layer III and terminate in middle (deep III to upper V) layers (“feedforward” pattern). Connections between areas of comparable laminar structure are issued from superficial (II–III) and deep (V–VI) layers and terminate across all layers (“lateral” pattern) (reviewed in Garcia-Cabezas et al., 2019). It is important to note that laminar patterns of connections between cortical areas are studied in laboratory animals with tract-tracing techniques (e.g., Barbas, 1986) in experiments that are precluded in humans for ethical reasons. These studies with neural tracers have confirmed the validity of the Structural Model for all the areas across the cerebral cortex and all the mammalian species studied so far (reviewed in Garcia-Cabezas et al., 2019). Thus, the cortical type analysis of the human cerebral cortex summarized in Tables 4–7 and represented in the maps of Figure 8 will allow for predictions of laminar patterns of corticocortical connections in humans based on the Structural Model (Barbas and Rempel-Clower, 1997; Garcia-Cabezas et al., 2019). For instance, we can advance that the laminar pattern of connections between two Dysgranular areas, like FL_2_ in the ACC and area LD in the retrosplenial cortex, will be lateral. In contrast, connections form Agranular area LA_1_ in the ACC to Eulaminate II area FD*_Δ_* in the DLPFC will be feedback; in the reverse direction, from area FD*_Δ_* in the DLPFC to area LA_1_ in the ACC, laminar patterns of connections will be feedforward.

Laminar patterns of connections are related to the direction of the flow of information across cortical areas and, therefore, with cortical hierarchies (Felleman and Van Essen, 1991; Mesulam, 1998; Hilgetag and Goulas, 2020). Modern imaging studies show gradients of cortical processing from primary to transmodal areas (Huntenburg et al., 2018; Paquola et al., 2019), in parallel to gradients of laminar differentiation and the laminar patterns of connections predicted by the Structural Model (Barbas and Rempel-Clower, 1997; Garcia-Cabezas et al., 2019). Thus, the cortical type analysis of the Atlas of von Economo and Koskinas (1925/2008) presented in this protocol paper will also allow for predictions of the position of human neocortical areas across cortical hierarchies (e.g., Zhang et al., 2020). For instance, connections between anterior and posterior cingulate areas in the human cortex will be lateral, but connections from ACC to DLPFC will be feedback; in the reverse direction, from DLPFC to ACC will be feedforward. These predictions will be useful for elaboration of models of cortical function and consciousness (e. g., Chanes and Barrett, 2016).

Cortical thickness, a structural feature of the human cerebral cortex that is frequently measured in imaging studies (e.g., Fischl and Dale, 2000; Scholtens et al., 2015; Wagstyl et al., 2020) and has been associated with functional features of cortical areas in some studies (Mahjoory et al., 2020), does not seem to be related to cortical type (Figure 8G). Some studies describe increasing cortical thickness across sensory processing hierarchies, primarily driven by layers III, V, and VI, and the opposite pattern across motor-frontal areas (Wagstyl et al., 2020), but the analysis was limited to Eulaminate areas across sensory-motor gradients and excluded limbic (Agranular and Dysgranular) areas. The study of Mahjoory et al. (2020) concluded that cortical thickness represents a proxy of cortical hierarchical level, but in this study limbic areas were not analyzed. A systematic comparison of cortical thickness and resting-state recordings across cortical gradients including Agranular and Dysgranular areas is needed to confirm the absence of correlation between cortical thickness and cortical type, and, therefore, with cortical hierarchies.

### Relation of Cortical Type to Synaptic Plasticity and Neuron Stability

Structural features of the neocortex related to neuron stability vary systematically in parallel to laminar elaboration. For instance, the content of intracortical myelin, a cellular feature that provides stability to neurons by facilitating the propagation of action potentials (Bishop, 1965) and also inhibits synaptic plasticity (Boghdadi et al., 2017), increases progressively in parallel to laminar elaboration. Actually, the intracortical content of myelin measured by MRI (T1w/T2w mapping) can be used as a proxy for laminar elaboration of neocortical areas (Huntenburg et al., 2017), as corroborated here (Figures 8D,E). In general, the better elaborated the laminar architecture of a given cortical area, the higher the intracortical myelin content of this area (Sanides, 1964, 1970; Barbas and Pandya, 1987, 1989; Barbas and García-Cabezas, 2015; Garcia-Cabezas et al., 2017; Royer et al., 2020), but it should be noted that there are some exceptions to this rule; for instance, posterior orbital areas are Agranular and Dysgranular but have higher content of intracortical myelin than expected for their cortical type (Barbas and Pandya, 1989; Zikopoulos et al., 2018), also seen using T1w/T2w ratio in MRI studies (Glasser and Van Essen, 2011).

The expression of perineuronal nets, another stabilizer of neurons, is also higher in neocortical areas with better laminar elaboration (Garcia-Cabezas et al., 2017). In contrast, the expression of markers that favor synaptic plasticity is higher in Agranular and Dysgranular areas of the prefrontal cortex in the macaque (Garcia-Cabezas et al., 2017). All these data suggest that neocortical areas with simpler laminar elaboration are more plastic and less stable than Eulaminate areas. Thus, the cortical type analysis of the Atlas of von Economo and Koskinas (1925/2008) summarized in Tables 4–7 and represented in the maps of Figure 8 will allow for predictions of differences in synaptic plasticity across human neocortical areas.

### Relation of Cortical Type to Selective Vulnerability

Post-mortem studies of human brains show that cortical areas with the simplest laminar structure are more vulnerable to neurological and neurodevelopmental disorders compared to areas with better laminar elaboration (Arnold et al., 1991; Braak and Braak, 1991; Duyckaerts et al., 1998; Zikopoulos and Barbas, 2010; Vismer et al., 2015; Burt et al., 2018; Zikopoulos et al., 2018). We suggested that factors that favor synaptic plasticity make Agranular and Dysgranular neocortical areas more flexible but also more vulnerable to disease: higher synaptic plasticity implies higher metabolic activity and cellular stress (Garcia-Cabezas et al., 2017).

Thus, the cortical type analysis of the Atlas of von Economo and Koskinas (1925/2008) summarized in Tables 4–7 and represented in the maps of Figure 8 will allow for predictions of differences in selective vulnerability across human neocortical areas. For instance, we hypothesize that the laminar pattern of dissemination of phosphorylated tau protein in Alzheimer’s disease (Arnold et al., 1991; Braak and Del Tredici, 2015) and other tauopathies across neocortical areas will be predicted by cortical type under the rules of the Structural Model (Barbas and Rempel-Clower, 1997; Garcia-Cabezas et al., 2019).

### Implications of Cortical Type Analysis for Developmental Studies

Recent developmental observations in human embryos show that Agranular and Dysgranular areas develop earlier than Eulaminate areas (Barbas and García-Cabezas, 2016; Garcia-Cabezas et al., 2019). Regarding neuron production, preliminary data in human fetuses show that embryonic layers under prospective Eulaminate areas have more mitotic activity than under Agranular and Dysgranular areas (Reillo et al., 2011). These data suggest that cortical types emerge in development as a result of differences in the windows of neurogenesis and neuron migration across the developing neocortex. The cortical type analysis of the human neocortex presented in this protocol paper will provide the framework for further research in the development of neocortical areas of different types to identify different windows of vulnerability to maldevelopment and disruption at genetic and epigenetic levels (García-Cabezas et al., 2018; Charvet, 2020).

## Conclusion: A Useful Tool for Studies in the Human Cerebral Cortex

In this protocol paper we summarize the principles of cortical type analysis to provide researchers with methods and criteria for defining sectors across the continuum of laminar gradients in the human neocortex, exemplified in the Atlas of von Economo and Koskinas (1925/2008). The systematic variation of laminar structure in gradients across neocortical areas is a fundamental feature of the human cerebral cortex rooted in development and evolution. Laminar variation across neocortical areas is related to cortical connectivity and hierarchical processing and underlies differences in synaptic plasticity and selective vulnerability (Garcia-Cabezas et al., 2019). Cortical type analysis as presented in this protocol paper will help spur and guide future research on the evolution, development, connectivity, synaptic plasticity, and selective vulnerability of the human cerebral cortex.

## Data Availability Statement

The raw data supporting the conclusions of this article will be made available by the authors, without undue reservation.

## Ethics Statement

The studies involving human participants were reviewed and approved by Institutional Review Board of Boston University (X3408). Written informed consent for participation was not required for this study in accordance with the national legislation and the institutional requirements.

## Author Contributions

MG-C and BZ designed the experiments. MG-C, JH, and BZ sliced and photographed the human brains used and analyzed Nissl stained sections of the human cerebral cortex and the scans from von Economo and Koskinas (1925/2008). JH cut the blocks containing prefrontal areas and stained sections for Nissl. MG-C and JH photographed Nissl stained sections of the human cerebral cortex. MG-C prepared the figures. BZ secured the funding. All authors contributed to writing the manuscript and approved its final version.

## Conflict of Interest

The authors declare that the research was conducted in the absence of any commercial or financial relationships that could be construed as a potential conflict of interest.
